# Supplementary material for: Increased Inflammation as well as Decreased Endoplasmic Reticulum Stress and Translation Differentiate Pancreatic Islets of Pre-symptomatic Stage 1 Type 1 Diabetes and Non-diabetic Cases
Source: bioRxiv. 2024 Sep 19:2024.09.13.612933. Preprint. [Version 1] doi: 10.1101/2024.09.13.612933 (PMC11429719; doi:10.1101/2024.09.13.612933)

bioRxiv preprint doi: https://doi.org/10.1101/2024.09.13.612933; this version posted September 19, 2024. The copyright holder for this preprint (which was not certified by peer review) is the author/funder, who has granted bioRxiv a license to display the preprint in perpetuity. It is made available under aCC-BY 4.0 International license.

Supplementary Information:

Supplemental Data 1.

Supplemental Tables 1-6 in excel spreasheets including the list of total identified islet proteins, significantly altered proteins, and the list of all quantified proteins in human islets.

Supplemental Figure 1.

Multiplex immunofluorescence imaging of islet proteins. (A) The antibody staining workflow; (B) Images of individual channel and merged image showing that NPTX2 is mainly expressed in beta cells within islets.

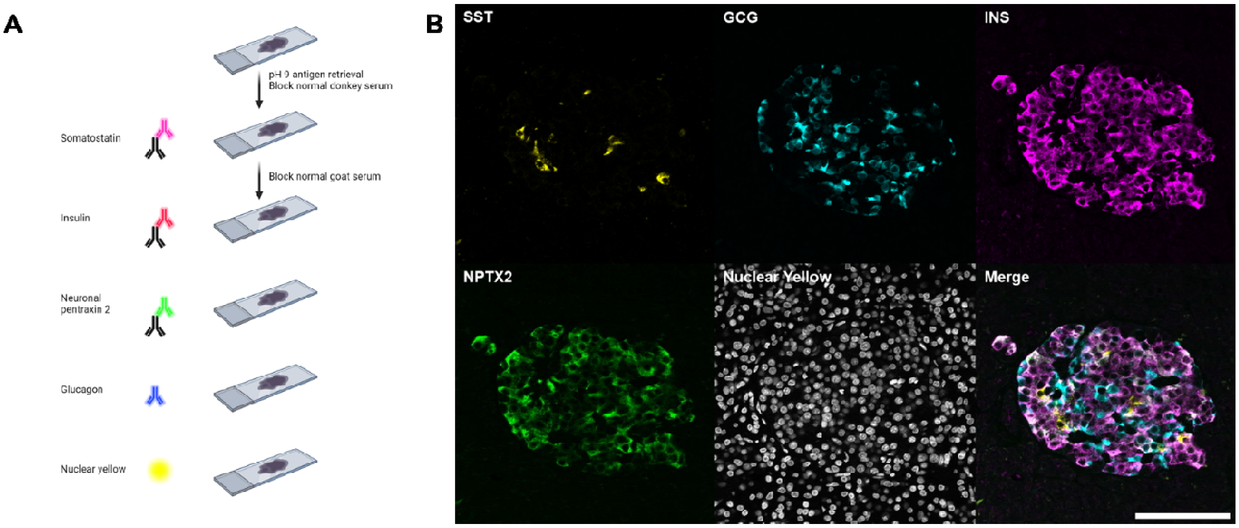

bioRxiv preprint doi: https://doi.org/10.1101/2024.09.13.612933; this version posted September 19, 2024. The copyright holder for this preprint (which was not certified by peer review) is the author/funder, who has granted bioRxiv a license to display the preprint in perpetuity. It is made available under aCC-BY 4.0 International license.

Supplemental Figure 2.

Revigo visualization of ontology of up- and down-regulated biological processes. The size of the circle indicates the number of proteins identified in the process. The vertical position indicates the uniqueness of the pathway with the top being more common, while the signficance of the pathway (log size) increases to the right.

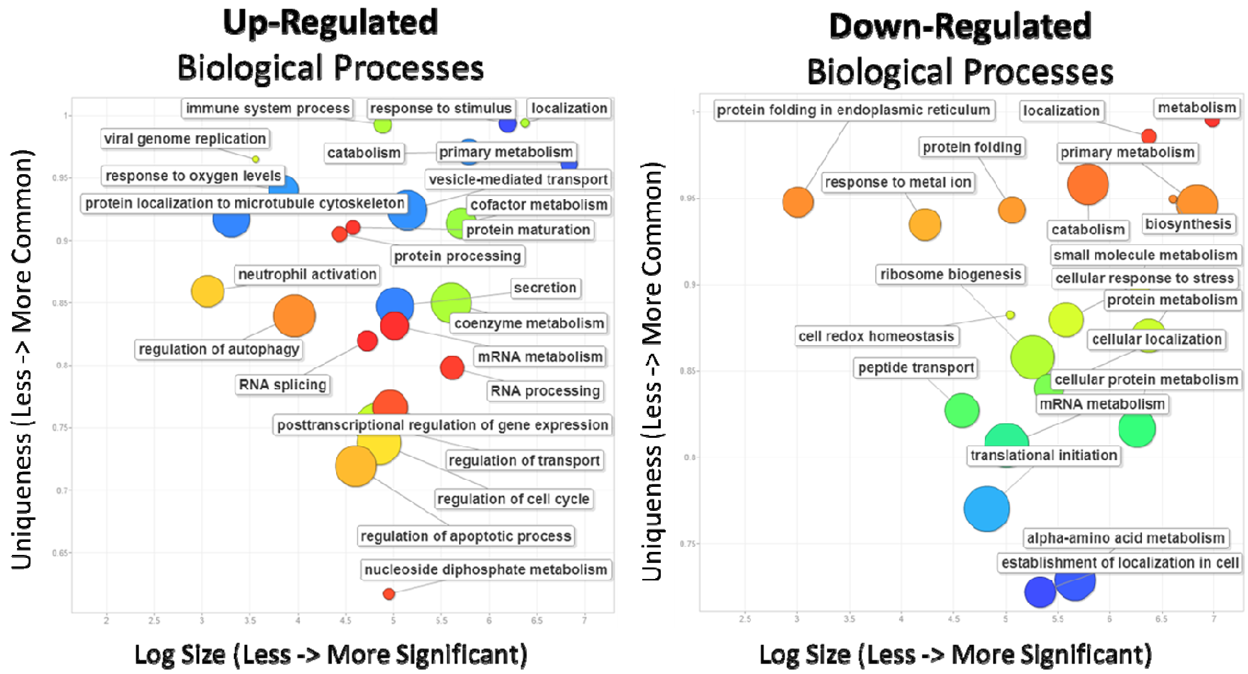

Supplement: Supplement 2 [file NIHPP2024.09.13.612933v1-supplement-2.pdf]
